# An administrative data merging solution for dealing with missing data in a clinical registry: adaptation from ICD-9 to ICD-10

**DOI:** 10.1186/1471-2288-8-1

**Published:** 2008-01-23

**Authors:** Danielle A Southern, Colleen M Norris, Hude Quan, Fiona M Shrive, P Diane Galbraith, Karin Humphries, Min Gao, Merril L Knudtson, William A Ghali, the APPROACH Investigators

**Affiliations:** 1 Department of Community Health Sciences, University of Calgary, Calgary, AB, Canada; 2 Department of Medicine, University of Calgary, Calgary, AB, Canada; 3 Centre for Health and Policy Studies, University of Calgary, Calgary, AB, Canada; 4 Faculty of Nursing, University of Alberta, Edmonton, AB, Canada; 5 Department of Medicine, University of British Columbia, Vancouver, BC, Canada; 6 Provincial Health Services Authority, Vancouver, BC, Canada

## Abstract

**Background:**

We have previously described a method for dealing with missing data in a prospective cardiac registry initiative. The method involves merging registry data to corresponding ICD-9-CM administrative data to fill in missing data 'holes'. Here, we describe the process of translating our data merging solution to ICD-10, and then validating its performance.

**Methods:**

A multi-step translation process was undertaken to produce an ICD-10 algorithm, and merging was then implemented to produce complete datasets for 1995–2001 based on the ICD-9-CM coding algorithm, and for 2002–2005 based on the ICD-10 algorithm. We used cardiac registry data for patients undergoing cardiac catheterization in fiscal years 1995–2005. The corresponding administrative data records were coded in ICD-9-CM for 1995–2001 and in ICD-10 for 2002–2005. The resulting datasets were then evaluated for their ability to predict death at one year.

**Results:**

The prevalence of the individual clinical risk factors increased gradually across years. There was, however, no evidence of either an abrupt drop or rise in prevalence of any of the risk factors. The performance of the new data merging model was comparable to that of our previously reported methodology: c-statistic = 0.788 (95% CI 0.775, 0.802) for the ICD-10 model versus c-statistic = 0.784 (95% CI 0.780, 0.790) for the ICD-9-CM model. The two models also exhibited similar goodness-of-fit.

**Conclusion:**

The ICD-10 implementation of our data merging method performs as well as the previously-validated ICD-9-CM method. Such methodological research is an essential prerequisite for research with administrative data now that most health systems are transitioning to ICD-10.

## Background

We have previously developed and reported on a method for dealing with missing data in a prospective cardiac registry database [1]. The method involves linking the prospectively-derived cardiac registry data on a patient-by-patient basis to corresponding administrative data, followed by a process of mapping the specific clinical diagnoses present in both the registry data and administrative data to create a single 'final' record of baseline diagnoses present in a given patient. Advantages of the methodology that we developed and validated are that it is conceptually simple (relative, perhaps, to more complex multiple imputation procedures), it can be readily implemented in many jurisdictions, it can be applied to non-random missing data situations such as ours, and it produces a 'complete' dataset that can then be used in subsequent statistical procedures for which data completeness is crucial. Since its initial description in the literature, the method has gone on to be widely used, both for research conducted by our group working with data from the Alberta Provincial Project for Outcome Assessment in Coronary Heart Disease (APPROACH) registry [2-4], and by other groups conducting similar research in other settings [5,6].

Our previously-described method was derived using a coding algorithm based on the 9^th ^revision of the International Classification of Diseases, Clinical Modification (ICD-9-CM) for mapping clinical diagnoses in administrative data [1]. The coding algorithm adopted elements of the Deyo ICD-9-CM adaptation of the Charlson comorbidity index for administrative data. We now, however, face a new methodological challenge as much of the world has since introduced the 10^th ^revision of the International Classification of Diseases and Related Health Problems (ICD-10).

The Tenth Revision (ICD-10) differs from the Ninth Revision (ICD-9 and ICD-9-CM) in several ways, although the overall content is similar: First, ICD-10 has alpha-numeric categories rather than numeric categories. Second, some chapters have been rearranged, some titles have changed, and conditions have been regrouped. Third, ICD-10 has almost twice as many categories as ICD-9. Fourth, some fairly minor changes have been made in the coding rules for mortality [7]. ICD-10 classifies a broader collection of diseases, injuries and causes of death, as well as external causes of injury and poisoning. Unlike ICD-9, ICD-10 applies beyond acute hospital care, and includes a broader collection of conditions and situations that are not diseases, but rather risk factors to health, such as occupational and environmental factors, lifestyle and psycho-social circumstances. ICD-10 is also more adaptable than previous versions, allowing more readily for the addition of codes as new diseases are discovered [8]. As a result of these enhancements, ICD-10 represents the broadest scope of any previous ICD revision to date.

ICD-10 was adopted in our province, Alberta, Canada, on April 1, 2002, and has been adopted by other Canadian provinces in recent years. Many other parts of the world such as Australia and much of continental Europe have also been using ICD-10 for the past several years. Because of this widespread ICD-10 uptake, there is now a pressing need to 'translate' existing ICD-9 methodological tools to corresponding tools for ICD-10. The process of translating ICD-9-CM algorithms into ICD-10 is far from straight forward, as many codes are not directly convertible from one version to the other. Furthermore, it is insufficient to use existing automated cross-walk algorithms, because many existing ICD-9 coding sequences have more than one corresponding coding section in ICD-10. Recently, Quan et al. published ICD-10 coding algorithms to define both Charlson and Elixhauser comorbidities [9], and the methodological process outlined in that work demonstrates the complexity of the ICD-9 to ICD-10 translation process.

Here we describe the derivation and evaluation of an ICD-10 coding algorithm for implementation of the ICD-9-CM data merging solution first proposed by Norris et. al. in our group [1]. Specifically, we present (1) the ICD-10 code selection process, (2) its implementation in the data merging process, and (3) its validation. To validate the methodology, we (a) compared the prevalences of specific clinical risk factors when implemented in ICD-9-CM and later in ICD-10, (b) modelled the 1-year mortality with clinical risk factors as predictors and (c) assessed how well the ICD-10 algorithm predicts mortality.

## Methods

### APPROACH Project

The Alberta Provincial Project for Outcome Assessment in Coronary Heart Disease (APPROACH) is a province-wide inception cohort of all adult Alberta residents undergoing cardiac catheterization for ischemic heart disease [10]. APPROACH was initiated to study provincial outcomes of care and facilitate quality assurance/quality improvement for patients with coronary artery disease in Alberta. The APPROACH database contains detailed clinical information on adult patients with known or suspected coronary artery disease (CAD) who undergo invasive cardiac procedures. Patients in APPROACH are followed longitudinally after cardiac catheterization, thus allowing for assessment of subsequent procedure use (i.e. percutaneous coronary intervention [PCI] or coronary artery bypass graft surgery [CABG]), as well as outcomes such as mortality and quality of life. Data collection is ongoing, and as is typical in prospective data registries, there are occasionally data fields that are not completed in the data collection process.

## Clinical variables

For the purposes of this data merging methodology research, clinical data were obtained for 86,649 adults (age ≥ 16 years) undergoing cardiac catheterization at one of the three hospitals in Alberta performing this procedure. Data elements recorded in the registry include patients' age, sex and presence of the following risk factors: cerebrovascular disease, congestive heart failure, chronic pulmonary disease, renal disease, type 1 diabetes, type 2 diabetes, dialysis, hyperlipidemia, hypertension, liver/gastrointestinal disease, malignancy, prior coronary artery bypass graft surgery, prior angioplasty, prior lytic therapy, prior myocardial infarction, and peripheral vascular disease. Clinical indication for catheterization is also collected at time of catheterization. It is these clinical variables that are occasionally missing in the data records produced for individual patients.

### Administrative data source

We obtained corresponding administrative data for all patients undergoing cardiac catheterization at the three Alberta hospitals performing the procedure. Administrative data records were selected for use when the admission and discharge dates in the administrative data encompass the date of cardiac catheterization recorded in APPROACH. These data are coded according to the ICD-9-CM for April 1 1995 – Mar 31 2002 (fiscal years 1995 through 2001), and coded according to the ICD-10 for April 1 2002 – Mar 31 2006 (fiscal years 2002 through 2005). A fiscal year runs from April 1^st ^of the calendar year through March 31^st^, and the fiscal year is designated according to the start years (i.e., fiscal year 2004 runs from April 1^st ^2004 through March 31^st ^2005). Follow-up started from time of catheterization and ran until Mar 31 2007, ensuring that all patients had at least 1 year of complete follow-up. Hospitals are required to submit discharge abstracts to the provincial Ministry of Health and the Canadian Institute for Health Information for each acute care hospital separation (discharge, transfer, or death) and for major outpatient procedures. Data elements acquired from the administrative data source included the patients' unique provincial personal health care number, the hospital chart number, sex, birth date, admission date, up to 16 diagnostic codes, and up to 10 procedure codes.

## APPROACH Methods

Our team has previously described our data merging methodology for dealing with missing data in APPROACH^1^. We developed ICD-9-CM coding definitions for each of the clinical variables identified in APPROACH, based largely on the ICD-9-CM comorbidity coding scheme derived by Deyo et. al. for defining the Charlson comorbidity index in American administrative data [11], and since then widely used by health services researchers in Canada and elsewhere. For variables that could not be matched to the Deyo coding algorithm in our originally described method [1], we scanned the ICD-9-CM for clinically appropriate codes to define specific clinical variables – a process implemented by two APPROACH team members (WAG & CMN), and through consensus. SAS computer code queries each of the diagnosis and procedure code fields in ICD-9-CM administrative data, thus defining presence or absence of each of the comorbidities.

### Derivation of the ICD-10 algorithm

Our first step in the translation process to develop an ICD-10 conversion of our ICD-9-CM definitions was to determine which clinical comorbidities in APPROACH needed de novo ICD-10 definitions, as opposed to coding definitions that could be borrowed from published methodological research on ICD-10. Fortunately, important published work has recently been completed by collaborators in Australia, Canada and Switzerland who have developed and validated a translation from ICD-9-CM to ICD-10 of the Charlson comorbidity index (Deyo coding algorithm) and the Elixhauser comorbidity coding method [9]. This recently-published ICD-10 coding algorithm developed through a rigorous multi-step process permitted us to use the derived data definitions for defining presence or absence of some of the clinical diagnoses in the ICD-10 administrative data used in our merging process. For the other variables (see below) that are not included in the above-mentioned ICD-10 adaptation of the Charlson or Elixhauser methods, we proceeded with a multi-step consensus process for code selection.

A committee of several team members determined the de novo ICD-10 coding algorithm for the following clinical variables: diabetes type 1, diabetes type 2, current smoker, indication for catheterization, prior thrombolytic, prior CABG and prior PCI. Procedure codes from the Canadian Classification of Interventions (CCI) were used to define the clinical variables that related to performance of a specific procedure, while ICD-10 was used to define the diagnosis codes. The coding consensus process involved a total of eight individuals originating from both the APPROACH team based in Alberta, and collaborators working in a 'sister' initiative, the British Columbia Cardiac Registries. All members received a package of resource materials that included old ICD-9-CM codes previously used with detailed descriptions of all codes, the ICD-10 manual and printouts of an ICD-10 compact disk search for medical terms. The team then met face-to-face and slowly worked through the manual as a group until all the variables had been defined. Upon completion of the face-to-face consensus process, a list of selected codes was drafted and re-circulated to all participants for final verification and approval.

### Implementation of ICD-10 merge

After using SAS code to query the administrative data, we merged administrative and clinical databases by provincial personal health numbers and hospital ID. If a comorbid condition was present in either database, our merged (enhanced) data was considered to have that condition present. This means that the clinical variables defined in administrative data were used in two situations: 1) when data were missing in APPROACH, in which case the administrative data filled in a missing data 'hole', and 2) to recode (i.e., enhance) clinical variables that were coded as absent in APPROACH when they were coded as being present in the administrative data [13] (a justifiable recoding of variables given the uniformly high specificity of variables coded as present in administrative data). The multi-step data merging methodology is summarized in Box 1.

## Analysis

After completing the conversion of our ICD-9-CM definitions into code for use with ICD-10, we enhanced our clinical data with administrative data. We then visually compared the prevalence of the clinical risk factors for the ICD-9-CM and ICD-10 methods on a plot of prevalence by year. We also compared two logistic regression models predicting mortality at one year based on the presence of clinical diagnoses present at baseline: (1) A model predicting 1-year mortality based on the 1995–2001 merged data, using ICD-9-CM coding, and (2) a model predicting 1-year mortality based on the 2002–2005 merged data using ICD-10 coding. All clinical variables listed in Table 1, in addition to age and sex, were entered into the logistic models with indication for catheterization also modelled (categorized as: MI, unstable angina, stable angina, or other indications [reference group]). To assess model performance of the two data merging methods, we determined the C-statistic for each of the two models. The C-statistic corresponds to the area under the receiver operating characteristic (ROC) curve and is a measure of model discrimination. It has a maximum possible value of 1.0. A value of 0.5 corresponds to a model that has no ability to discriminate beyond chance. Ninety-five percent confidence intervals were calculated for the c-statistic using bootstrap. Lastly, we visually assessed the models' goodness-of-fit by plotting observed versus expected percentages across analysis-defined deciles of risk. The risk groups used in this latter graphical assessment of model performance were those that arose from the models described above. All statistical analyses were performed using SAS version 8.1 (Cary, NC).

**Table 1:** ICD-9-CM and ICD-10 coding scheme used to define variables in database

**Variables**	**ICD-9-CM code**	**ICD-10 code**
**Cerebrovascular disease**	430.x–438.x	G45.x, G46.x, H34.0, I60.x–I69.x
**Pulmonary (COPD)**	490.x–496.x, 500.x–505.x, 506.4	I27.8, I27.9, J40.x–J47.x, J60.x–J67.x, J68.4, J70.1, J70.3
**Congestive heart failure**	428.x	I09.9, I11.0, I13.0, I13.2, I25.5, I42.0, I42.5–I42.9, I43.x, I50.x, P29.0
**PVD**	441.x, 443.9, 785.4, V43.4	I70.x–I71.x, I73.1–I73.9, I77.1, I79.0, I79.2, K55.1, K55.8, K55.9, Z95.8, Z95.9
**Liver/GI**		
**Mild Liver Disease**	571.2, 571.5, 571.6, 571.4	B18.x, K70.0–K70.3, K70.9, K71.3–K71.5, K71.7, K73.x, K74.x, K76.0, K76.2–K76.4, K76.8, K76.9, Z94.4
**Peptic Ulcer Disease**	531x–534.x	K25–-K28.x
**Moderate Severe Liver Disease**	572.2, 572.3, 572.4, 572.8, 456.0–456.21	I85.0, I85.9, I86.4, I98.2, K70.4, K71.1, K72.1, K72.9, K76.5–K76.7
**Renal disease**	582.x, 583.x, 584.x, 585.x, 586.x, 588.x	I12.0, I13.1, N03.2–N03.7, N05.2–N05.7, N18.x, N19.x, N25.0, Z49.0–Z49.2, Z94.0, Z99.2
**Malignancy/Metastatic disease**	140.x–172.x, 174.x–208.x	C00.x–C26.x, C30.x–C34.x, C37.x–C41.x, C43.x, C45.x–C58.x, C60.x–C76.x, C77.x–C80.x, C81.x–C85.x, C88.x, C90.x–C97.x
**Hypertension**	401.x–405.x	I10.x, I11.x–I13.x, I15.x
**Hyperlipidemia**	272.0, 272.1, 272.2, 272.3, 272.4	E78.0–E78.5
**Dialysis**	V42.0, V45.1, V56.0, V56.1, V56.8, 39.27, 39.42, 39.93–39.95, 54.98	Z49.x, Z99.2 *Procedure codes*: 1.KY.76, 1.OT.53, 1.PZ.21
**Diabetes Type 1**	250.0 – 250.9 with 5^th ^digits 1 & 3	E10.x, E13.10, E13.12, E14.10, E14.12
**Diabetes Type 2**	250.0 – 250.9 with 5^th ^digits 0 & 2	E11.x, E13.0, E14.0
**Current Smoker**	305.10, 305.11, 305.12	F17.x, T65.2, Z50.8, Z71.6, Z72.0, P04.2 Procedure codes*: 5.AD.14.BK, 7.SP.10.VK
**Prior MI**	412.x	I25.2
INDICATION		
**MI**	410.x	I21.x–I23.x
**Unstable angina**	411.1, 413.0, 411.89, 411.81	I20.0, I24.x
**Stable angina**	413.1, 413.9	I20.8, I20.9, I25.0, I25.1, I25.8, I25.9
**Other**	None of the above codes for indication present	None of the above codes for indication present
**Prior CABG**	V45.81	Z95.1
**Prior PCI**	V45.82	Z95.5

* Procedure codes can by found in ICD-10-CA/CCI (Canadian Classification of Health Interventions)

## Summary of Methodology

1. Determine frequencies of missing data in clinical registry data;

2. Prepare clinical registry data with coding as 1 = present and 0 = absent. If missing or unknown, code as 0;

3. Obtain corresponding administrative data records with admission and discharge dates that encompass the procedure date;

4. Prepare administrative data with coding of conditions as 1 = present or 0 = absent based on coding algorithm;

5. Merge data from step 2 and step 4 by personal health number and/or hospital chart number;

6. Define final variables as 1 if present in either data from step 2 or step 4. Otherwise, code as 0;

7. Determine frequencies of final merged clinical variables by fiscal year.

## Results

### Coding Algorithm

The new ICD-10 coding algorithm developed by consensus is presented in Table 1, along with the original ICD-9-CM algorithm that we previously reported [1]. The ICD-10 coding algorithm provides several more codes than the ICD-9-CM algorithm with changes perhaps most notable in the coding approaches for stable and unstable angina.

### Pattern of missing data

We present the pattern of missing data in the original APPROACH dataset in Table 2. Overall there were 58.9% of cases with at least one variable missing. The proportion of missing data varies over the years, but the proportion of cases with at least some missing data remains fairly constant across years. For a two year period (2001 & 2002) missing values for prior CABG and prior PCI were automatically coded as "0" in the raw APPROACH data (as opposed to being coded as unknown or missing). This issue was recognized and adjusted such that rates of missing data for these two variables increased again in 2003. This issue did not affect any of the other study variables.

**Table 2:** Percentage (%) of cases with missing data for specific variables in the original APPROACH database

		**FISCAL YEAR**										
		**ICD-9-CM**							**ICD-10**			

**CLINICAL VARIABLES**	**TOTAL**	**1995**	**1996**	**1997**	**1998**	**1999**	**2000**	**2001**	**2002**	**2003**	**2004**	**2005**

**N**	*86,649*	*6,348*	*6,131*	*6,242*	*6,718*	*8,110*	*8,469*	*8,479*	*8,767*	*8,835*	*9,257*	*9,293*
**Dialysis (%)**	*11.5*	4.7	2.8	2.5	3.6	4.9	2.1	24.1	11.0	20.7	21.5	18.2
**Diabetes Type I (%)**	*6.5*	4.7	2.8	2.5	3.6	2.9	0.9	14.4	8.8	7.8	10.0	9.2
**Renal (Creatinine > 200 mmol/L) (%)**	*29.8*	20.8	12.7	15.3	28.8	59.5	43.2	32.3	26.7	25.1	24.6	30.0
**Malignancy/Metastatic Disease (%)**	*10.4*	7.0	5.7	5.9	5.9	5.1	2.1	20.5	17.7	14.4	11.7	13.5
**Prior CABG (%)**	*9.2*	20.8	11.1	10.4	10.9	12.2	6.3	1.1	0.1	10.0	12.4	10.3
**Liver/GI (%)**	*11.1*	0.2	0.1	5.4	5.9	5.1	2.1	39.1	16.4	13.2	11.3	13.8
**Prior PCI (%)**	*9.3*	19.3	11.3	10.1	11.0	12.7	6.6	1.2	0.2	10.0	12.8	12.1
**Cerebrovascular Disease (%)**	*10.8*	4.7	2.8	2.5	3.6	4.9	2.1	11.9	11.3	21.3	23.7	20.1
**PVD (%)**	*10.4*	4.7	2.8	2.5	3.6	4.9	2.1	12.0	11.3	21.4	21.0	18.6
**Pulmonary Disease (%)**	*9.3*	7.0	5.7	5.9	5.9	5.1	2.1	17.6	14.2	11.7	11.0	12.5
**Congestive heart failure (%)**	*14.1*	20.6	17.3	8.3	10.7	16.0	10.2	12.5	10.8	17.2	17.5	14.4
**Diabetes Type II (%)**	*6.7*	4.7	2.8	2.5	3.6	2.9	0.9	14.7	8.9	7.6	10.3	10.1
**Prior MI (%)**	*15.8*	19.3	15.9	9.5	12.2	16.6	11.4	17.7	18.2	19.1	17.5	14.9
**Hypertension (%)**	*11.2*	14.9	12.4	7.8	10.8	15.1	10.9	11.5	10.0	8.7	12.2	9.3
**Hyperlipidemia (%)**	*17.4*	28.0	26.8	19.9	20.8	22.7	17.1	14.8	12.9	13.2	12.1	11.0
**Proportion with one or more missing**	*58.9*	48.2	45.8	39.5	49.5	72.3	58.3	74.1	60.0	64.8	60.2	61.3

### Percentage of the clinical conditions

**Figure 1:**
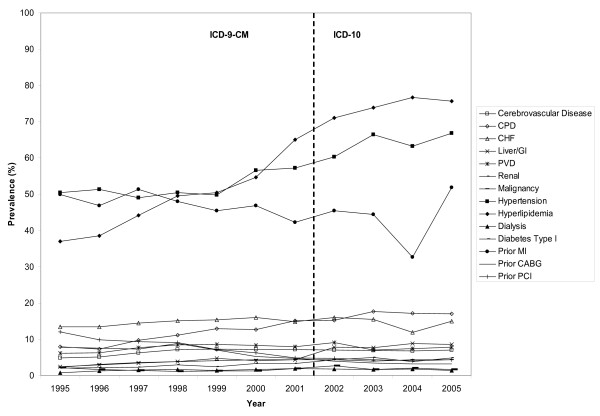
Percentage (%) of comorbidities defined by ICD-9-CM and ICD-10 coding schemes by year.

The data consisted of 86,649 patients who underwent cardiac catheterization between April 1, 1995 and March 31, 2006. Table 3 provides the percentage of clinical conditions derived from the ICD-9-CM coding method using 1995–2001 data and the ICD-10 coding method using 2002–2005 data. The percentage of the individual clinical conditions tended to increase linearly for most conditions (Figure 1). Importantly, however, there is no concerning jump (or drop) in the percentages at the time of the change from ICD-9-CM to ICD-10.

**Table 3:** Percentage (%) of clinical comorbidities of enhanced data from APPROACH

		**FISCAL YEAR**										
		**ICD-9-CM**							**ICD-10**			

**VARIABLE**	**TOTAL**	**1995**	**1996**	**1997**	**1998**	**1999**	**2000**	**2001**	**2002**	**2003**	**2004**	**2005**

**N**	*86,649*	6,348	6,131	6,242	6,718	8,110	8,469	8,479	8,767	8,835	9,257	9,293
**Mean Age (Std. Dev.)**	*62.6 (12.0)*	62.0 (11.4)	62.6 (11.5)	62.7 (11.4)	62.7 (11.7)	62.7 (12.1)	62.6 (12.1)	62.6 (12.0)	62.6 (12.3)	62.6 (12.5)	62.5 (12.4)	63.1 (12.4)
**Percent > 75 years**	*16.6*	12.4	14.4	14.7	15.1	17.0	17.0	17.0	17.3	17.8	18.5	18.9
**Percent Male**	*69.4*	70.2	70.6	69.7	70.5	68.4	69.6	68.2	69.7	68.8	69.6	69.2
**Comorbidities**												
**Dialysis**	*1.6*	0.8	1.3	1.5	1.7	1.4	1.7	1.9	1.8	1.7	1.8	1.4
**Diabetes Type I**	*1.8*	2.2	1.7	1.4	1.1	1.3	1.3	1.9	2.7	1.7	2.0	1.7
**Renal (Creatinine > 200 mmol/L)**	*3.5*	2.0	2.2	2.3	2.9	2.4	3.3	3.3	4.4	5.0	3.9	4.9
**Malignancy**	*4.0*	2.3	3.1	3.6	3.8	4.1	4.4	4.3	4.4	4.0	4.4	4.4
**Prior CABG**	*5.4*	7.8	7.5	7.3	8.8	7.1	5.2	4.8	4.0	3.5	3.2	3.2
**Liver/GI**	*5.7*	2.4	3.0	3.5	3.9	4.7	4.1	4.4	7.8	7.7	8.8	8.6
**Prior PCI**	*6.5*	12.0	9.8	9.3	9.1	7.2	6.3	4.9	4.8	4.3	4.1	4.3
**Cerebrovascular Disease**	*6.7*	4.9	5.1	6.3	7.2	7.3	7.2	7.2	7.1	6.9	6.8	7.0
**PVD**	*7.8*	6.1	6.3	7.7	8.4	8.6	8.3	7.9	9.1	7.1	7.4	7.8
**COPD**	*13.6*	8.0	7.3	9.7	11.1	12.9	12.7	15.1	15.2	17.7	17.2	17.0
**Congestive heart failure**	*14.7*	13.4	13.5	14.5	15.1	15.4	16.0	14.9	16.0	15.5	11.9	15.0
**Diabetes Type II**	*19.8*	15.4	15.4	18.3	18.2	18.3	19.4	20.3	21.5	22.4	21.3	23.1
**Current Smoker**	*26.5*	25.4	23.7	26.0	25.1	25.9	27.9	27.6	26.8	26.9	27.1	27.8
**Prior MI**	*45.5*	50.0	46.9	51.3	48.0	45.4	46.9	42.3	45.4	44.4	32.7	51.8
**Hypertension**	*57.4*	50.4	51.4	49.0	50.4	49.8	56.6	57.2	60.3	66.4	63.2	66.8
**Hyperlipidemia**	*59.9*	37.0	38.5	44.2	49.5	50.4	54.7	65.0	71.1	73.9	76.7	75.7
**INDICATION FOR CATH**												
**Stable Angina**	*31.4*	29.4	30.9	29.7	31.0	29.1	27.6	28.2	34.0	34.1	33.7	35.3
**Unstable Angina**	*25.6*	31.7	29.1	27.5	26.2	26.4	27.8	26.2	24.2	24.5	27.5	14.3
**MI**	*31.6*	21.0	23.7	30.8	29.0	28.1	30.9	32.3	35.0	35.0	31.7	43.2
**Other**	*11.5*	17.9	16.4	12.1	13.7	16.4	13.7	13.4	6.8	6.5	7.1	7.2

### Model performance for predicting mortality

The C-statistics for the two models predicting mortality based on the clinical variables were as follows: 0.788 (95% CI 0.775, 0.802) for the ICD-10 model versus c-statistic = 0.784 (95% CI 0.780, 0.790) for the ICD-9-CM model. These parameters suggest comparable performance in predicting mortality for the two models (ICD-10 and ICD-9-CM). Norris et al. previously demonstrated that the merged data performs better than the complete set of clinical variables without use of the administrative data merging methodology [1]. We find this to be true again for this analysis: c = 0.745 for the complete set of clinical variables without use of the administrative data merging versus c = 0.780 for the complete merged data. A subgroup analysis also showed that ICD10 performing slightly better than ICD-9-CM for an analysis stratified by clinical indication subgroup: For acute coronary syndrome (ACS) indication, ICD-10 c = 0.798 vs. ICD-9-CM c = 0.792; For non-ACS indication: ICD-10 c = 0.757 vs. ICD-9-CM c = 0.750.

**Figure 2:**
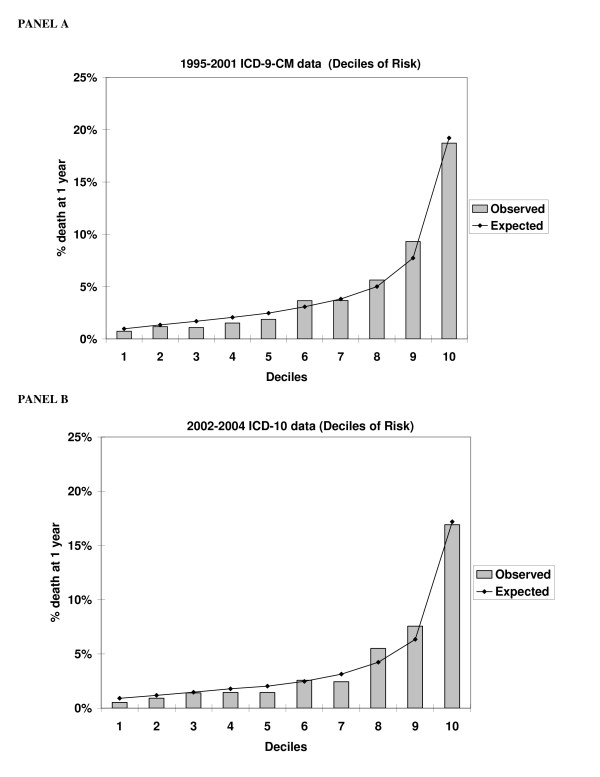
Observed and Expected in-hospital mortality by risk group for the ICD-9-CM coding method using 1995–2001 data (Panel A) and the ICD-10 coding method using 2002–2005 data (Panel B).

The observed and expected percentages of death at one year across decile-of-risk categories were plotted for each of the two methods (Figure 2). The ICD-9-CM coding method clearly predicts mortality and "spreads out" risk estimates, as does the ICD-10 coding method. The ICD-9-CM coding method estimates a spread of risk across decile groupings ranging from 0.7% for the lowest risk decile to 18.7% for the highest risk decile; the ICD-10 coding method produces a comparable spread of risks across deciles ranging from 0.5% to 16.9%.

## Discussion

In light of ICD-10 being introduced in 1992 by the World Health Organization, and being adopted by numerous countries internationally, researchers are now faced with the methodological challenge of developing ICD-10 coding algorithms for applied health services and population health research. ICD-10 is a new system with potential enhancement of coded information. For that potential to be fulfilled, however, there is a need for validated methodological tools for ICD-10. This paper contributes to the literature by demonstrating the process of taking a validated ICD-9-CM tool and adapting it (through a process of code translation) to a validated ICD-10 tool. Similar to the recent work of Quan et al. in this area [9], this type of research is an essential prerequisite for effective health research to proceed with administrative data.

With the substantial cost associated with producing clinical registries such as APPROACH, and the commonly associated challenge of missing clinical registry data, our data merging method and translation to ICD-10 can be of value to other researchers. As already mentioned, the methodology that we have described (both ICD-9-CM and ICD-10) is in use for applied research studies performed using APPROACH data, and is also being applied in other jurisdictions. A prerequisite for implementation of this data merging solution is, of course, that researchers need to gain access to available administrative data – a process that is not always simple in the context of health data privacy considerations and locally variable administrative data access procedures. Data access challenges aside, however, our data merging method has numerous advantages over other missing data solutions such as 'imputing to zero'(where a missing value is assumed to mean that the condition is absent), complete case analysis, or multiple imputation. Such alternate approaches bring the disadvantages of, respectively, occasionally lacking validity, losing cases, and introducing statistical complexity. It is such consideration of advantages and disadvantages that has led the APPROACH network to now routinely use the data merging method described here as its missing data solution of choice [12].

A limitation of our study is that our data merging algorithm may need modification for use in other clinical data registry initiatives if the baseline clinical variables collected do not map directly to those that we define in our coding algorithm. In instances where the coding algorithm and method are modified, we caution that validity assessments such as those presented here should be performed. A second caveat is that ICD-10 coding quality (relative to a gold standard assessment of presence/absence of specific conditions) may vary across jurisdictions, and that there may be a phenomenon of improved validity of ICD-10 coding over time as coders become more familiar with the new coding system. In relation to this latter point, it would have been highly informative to have a year of dually-coded data, with both ICD-9-CM and ICD-10 codes assigned to individual cases; such a data resource would have permitted a truer test of the comparability of the ICD-9-CM vs. ICD-10 coding algorithms. In this regard we emphasize that the logistic regression analysis that we did is not in and of itself proof of seamless transition and validation across algorithms. However, the logistic regression findings, in combination with the relatively smooth prevalence transitions presented in Table 3 suggest a reasonable performance. Interpretation of prevalence trends is challenging and somewhat difficult because of real clinical trends over time and also because of challenges such as evolving clinical definitions (e.g. the MI definition).

Despite these limitations, this paper presents the important methodological development work that health researchers need to undertake in coming years as a number of validated ICD-9-CM methodological tools for administrative data now need to be translated to ICD-10 so that productive and important health research can continue to be performed.

## Conclusion

In this instance, we demonstrate the successful translation of an ICD-9-CM coding algorithm to ICD-10 for dealing with missing data in a clinical registry initiative. Our work reveals a seamless transition to ICD-10 for our methodological tool, and provides a template for others to follow as they undertake similar work.

## Competing interests

The author(s) declare that they have no competing interests.

## Authors' contributions

DAS participated in conception and design, acquisition of data, analysis and interpretation of data and drafted the manuscript. CMN participated in the conception and design of the study and the acquisition of data. HQ, FMS, PDG, KH, MG, MLK, WAG participated in the conception and design of the study. FMS, PDG, MLK, WAG participated in the acquisition and interpretation of data. FMS and WAG helped draft the manuscript. All authors read and approved the final manuscript.

## Pre-publication history

The pre-publication history for this paper can be accessed here:


